# Supplementary material for: Synthetic biology-instructed transdermal microneedle patch for traceable photodynamic therapy
Source: Nat Commun. 2022 Oct 20;13:6238. doi: 10.1038/s41467-022-33837-1 (PMC9585024; doi:10.1038/s41467-022-33837-1)
Supplement: Supplementary file 1 — Supplementary Information [file 41467_2022_33837_MOESM1_ESM.pdf]

Supplementary Information

Synthetic biology-instructed transdermal microneedle patch for traceable photodynamic therapy

*Gang He[1], Yashi Li[1], Muhammad Rizwan Younis[1], Lian-Hua Fu[1], Ting He[1], Shan Lei[1], Jing Lin[1], and Peng Huang[1]**

[1]Marshall Laboratory of Biomedical Engineering, International Cancer Center, Laboratory of Evolutionary Theranostics (LET), School of Biomedical Engineering, Shenzhen University Health Science Center, Shenzhen 518060, China

(Email: peng.huang@szu.edu.cn)

Index:

Supplementary Figure 1 Synthesis and characterizations of CCPCA NPs 3
Supplementary Figure 2 CAT was loaded by CCPCA NPs 4
Supplementary Figure 3 Schematic of the NP-loaded MN patch prepared by a two-layer strategy 6
Supplementary Figure 4 The morphology of MN patches 7
Supplementary Figure 5 The MN-CCPCA patch provides sufficient strength to insert into the epidermal barrier 8
Supplementary Figure 6 CCPCA NPs maximize the accumulation of PpIX................................9
Supplementary Figure 7 Oxidative stress-mediated $[Ca]_{CYT}$ overload 10
Supplementary Figure 8 Lack of laser irradiation cannot induce oxidative stress 11
Supplementary Figure 9 Biosafety after the application of MN patches...................................12
Supplementary Figure 10 Ex vivo FL images of major organs and tumors in 4T1 tumor model after different administrations of 5-ALA and CCPCA 14
Supplementary Figure 11 Antitumor effects of MN-CCPCA patch in a 4T1 tumor model 15
Supplementary Figure 12 Ex vivo FL images of major organs and tumors in U87MG tumor model after different administrations of 5-ALA and CCPCA...16
Supplementary Figure 13 Antitumor effects of MN-CCPCA patch in a U87MG tumor model ..17
Supplementary Figure 14 H&E staining of tumor slices collected from different administration of 5-ALA and CCPCA groups 18
Supplementary Table 1. Accurate calculated value of tumor area, positive area ratio and positive area density of anti-GSH FL signals in 4T1/U87MG tumors by Aipathwell immunofluorescence analysis. 19

Supplementary Figures:

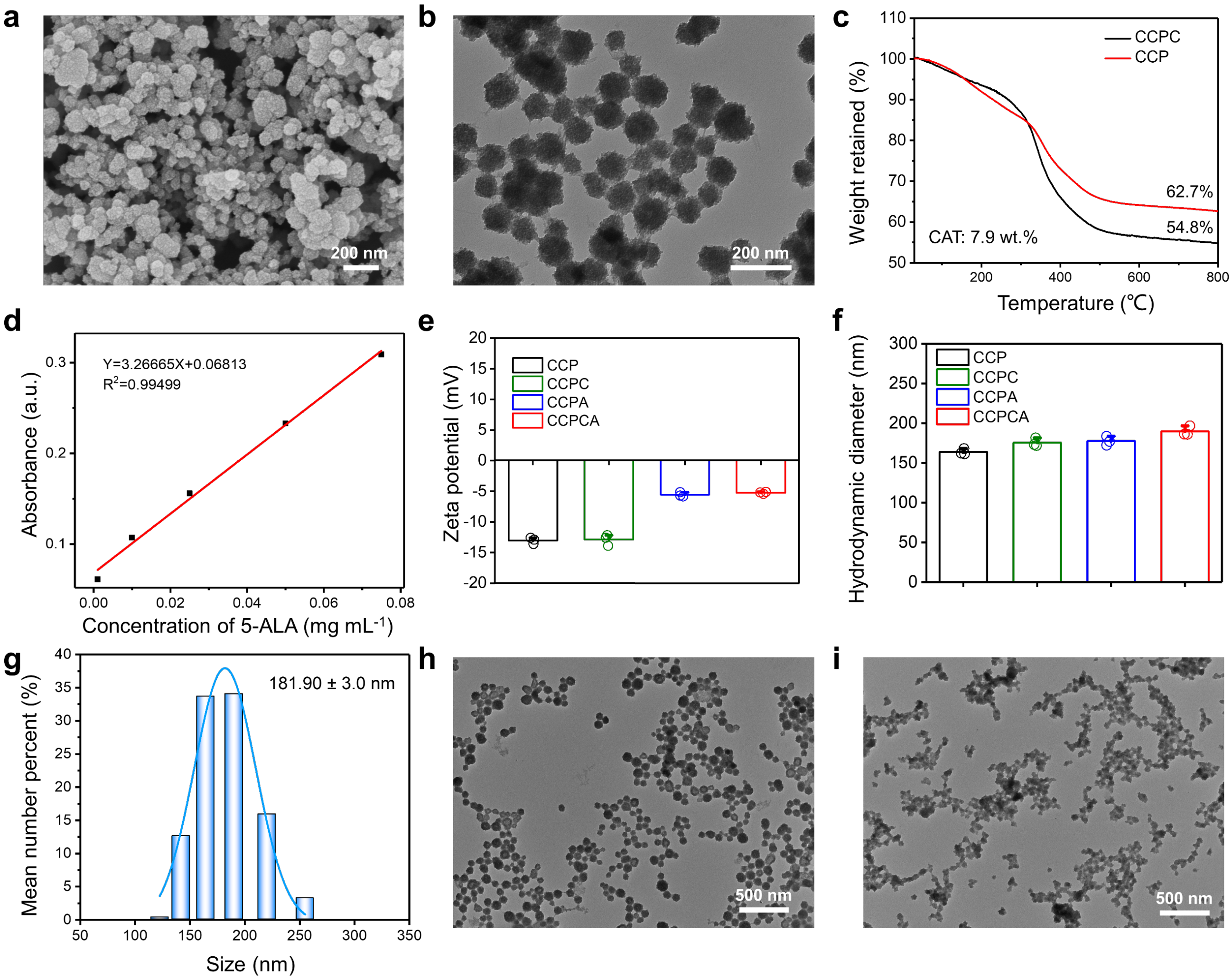

Supplementary Figure 1 Synthesis and characterizations of CCPCA NPs. (a) SEM images of CCPCA NPs and **(b)** TEM images of CCP NPs, scale bars = 200 nm. n = 3 independent experiments. **(c)** TGA analysis of CCP and CCPC NPs. n = 3 independent experiments. **(d)** The standard curve of UV-vis absorbance of 5-ALA aqueous solutions. **(e)** Zeta potential and **(f)** hydrodynamic diameter distribution of CCP, CCPA, CCPC and CCPCA NPs. Data represent the means ± SD (n = 3). **(g)** The mean number percent of the hydrodynamic size of CCPCA NPs. n = 3 independent experiments. TEM images of CCPCA NPs after soaking CCPCA NPs in PBS solutions at **(h)** pH 7.4 and **(i)** pH 6.0 for 4 h; scale bar = 500 nm. Images are from three independent samples, n = 3. Source data are provided as a Source Data file.

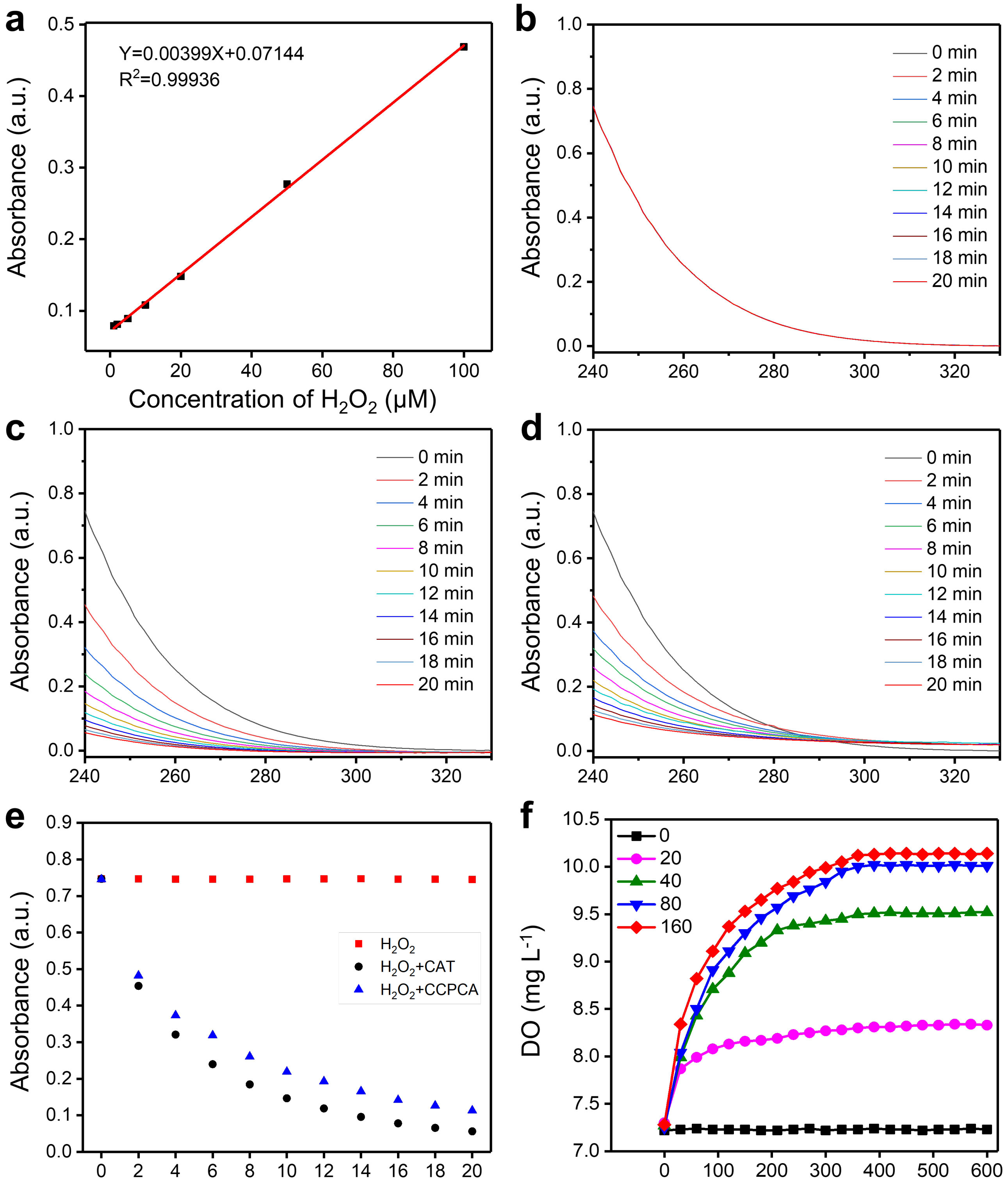

Supplementary Figure 2 CAT was loaded by CCPCA NPs. (a) The standard curve of UV-vis absorbance of H_2O_2 aqueous solutions and UV-vis absorption spectra of H_2O_2 after **(b)** deionized (DI) water, **(c)** 0.1 μg mL^{-1} CAT and **(d)** 1.2 μg mL^{-1} CCPCA NP treatment. n = 3 independent experiments. **(e)** The absorption of 10 mM H_2O_2 solution at 240 nm under the

indicated treatment conditions. n = 3 independent experiments. **(f)** The change of DO in H_2O_2 solution (100 μM) after adding CCPCA NPs at 0-160 μg mL^{-1}. n = 3 independent experiments. Source data are provided as a Source Data file.

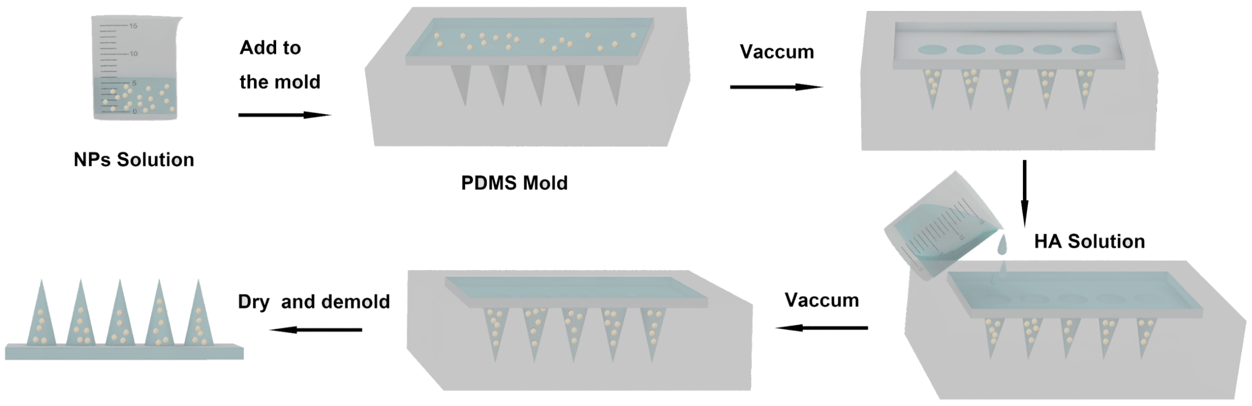

Supplementary Figure 3 Schematic of the NP-loaded MN patch prepared by a two-layer strategy. The NPs were dispersed in 600 μL DI water. Then, 100 μL NP suspension (containing 10 mg of NPs) was deposited on the surface of each PDMS mold through a pipette, and the solution was concentrated to the MN tips under vacuum for 3 min. The above deposition procedure was repeated at least five times. The whole device was then placed in a vacuum drying oven for to evaporate the water from the microneedle tip before HA solution (180 mg mL^{-1}, the weight to volume ratio of sodium hyaluronate to hyperactive hyaluronic acid was 1:5) was added to each PDMS mold surface. The final device was dried at 37 °C for 13 h and the MN patch was separated from the PDMS mold and stored in a dryer in the dark at room temperature. Source data are provided as a Source Data file.

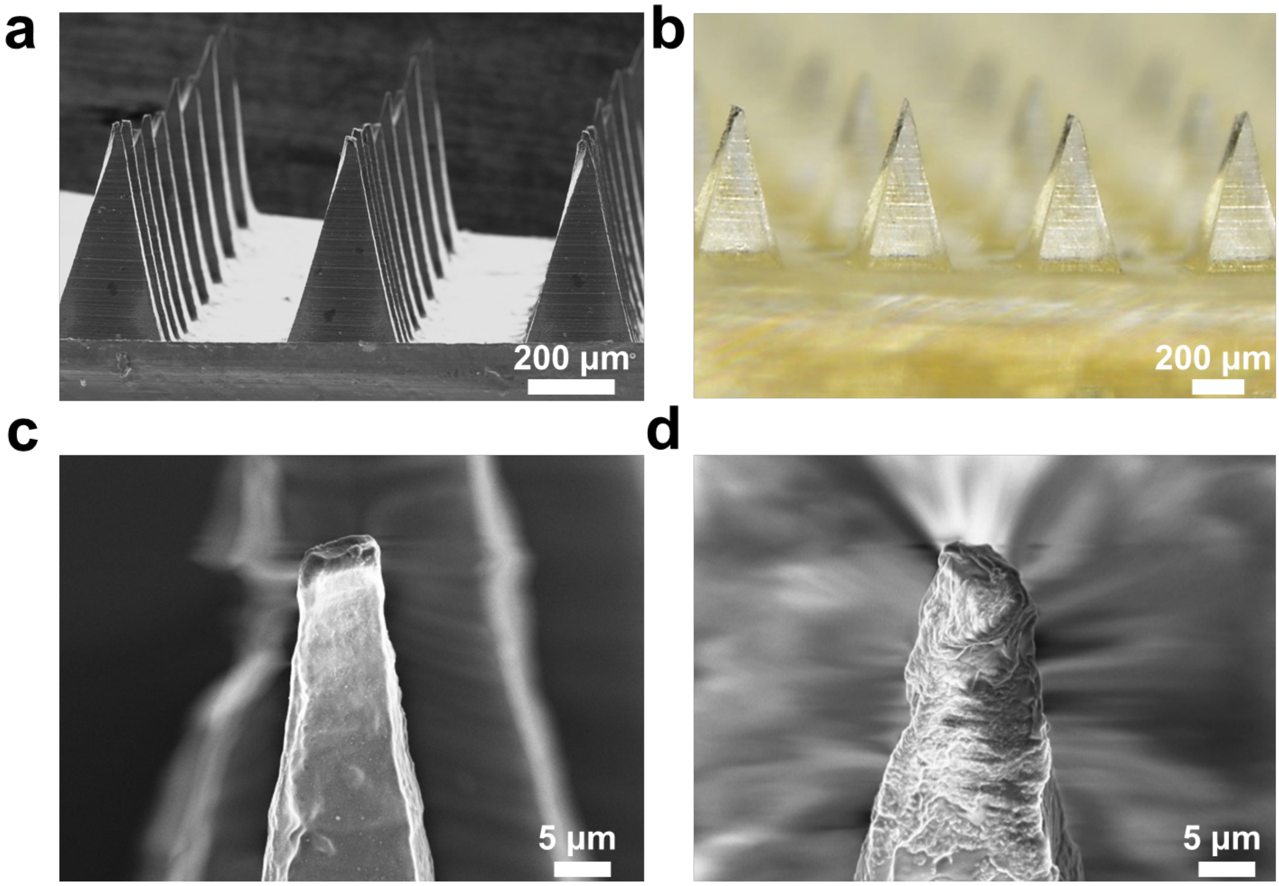

Supplementary Figure 4 The morphology of MN patches. Morphology of the MN patch before NP loading by **(a)** SEM and **(b)** stereo microscopy photographed at 90°, scale bar = 200 μm. The microneedle tip at high magnification, (**c**) before and (**d**) after loading CCPCA NPs, scale bar = 5 μm. Images are from three independent samples, n = 3. Source data are provided as a Source Data file.

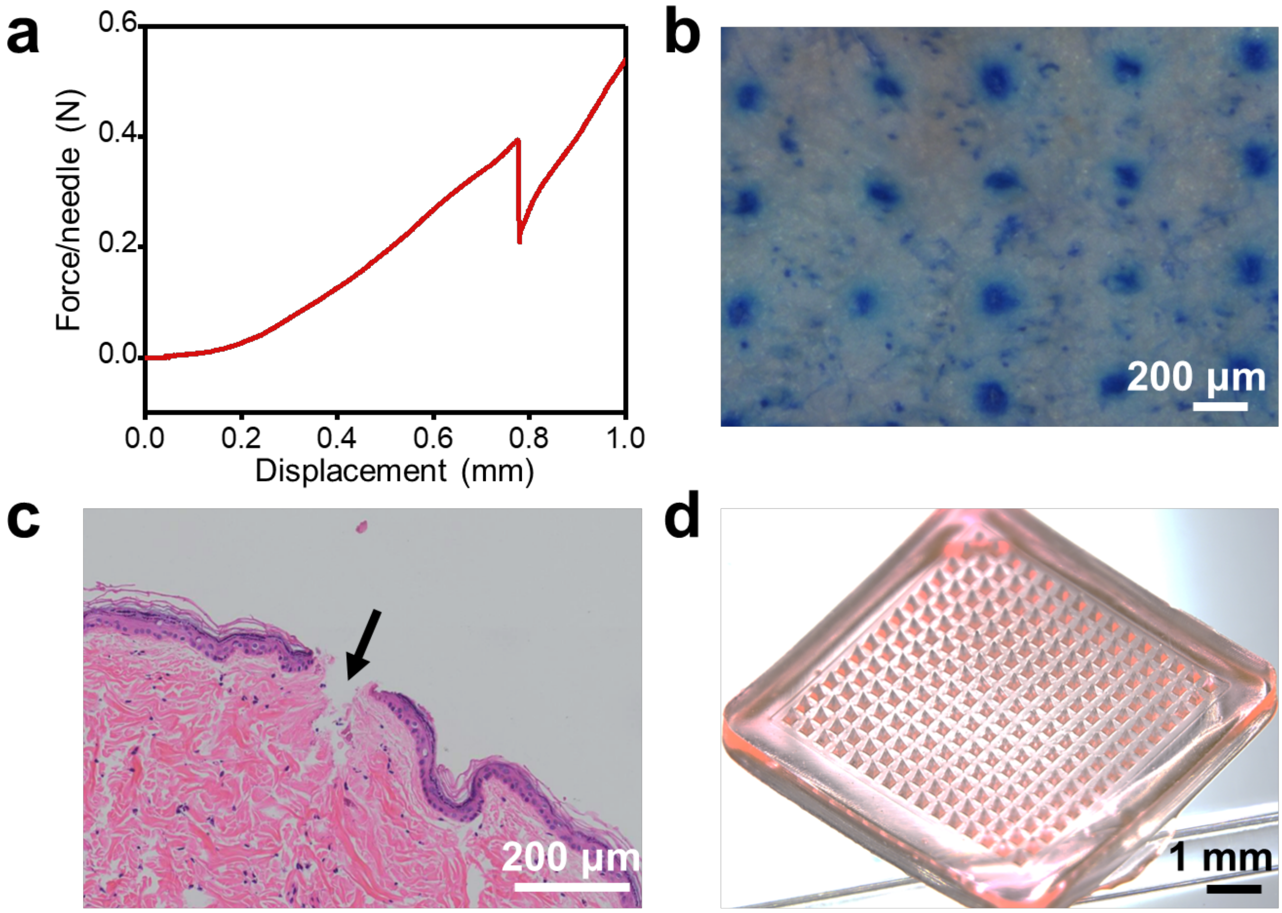

Supplementary Figure 5 The MN-CCPCA patch provides sufficient strength to insert into the epidermal barrier. (a) Mechanical property of MN-CCPCA patch. **(b)** MB staining and **(c)** H&E staining images of rat skin after application of MN-CCPCA patch, scale bar = 200 μm. Images are from three biologically independent skin samples of four-week-old female rats, n = 3. **(d)** Optical image of the MN patch containing CCPCA-RhB NPs, scale bar = 1 mm. n = 3 independent experiments. Source data are provided as a Source Data file.

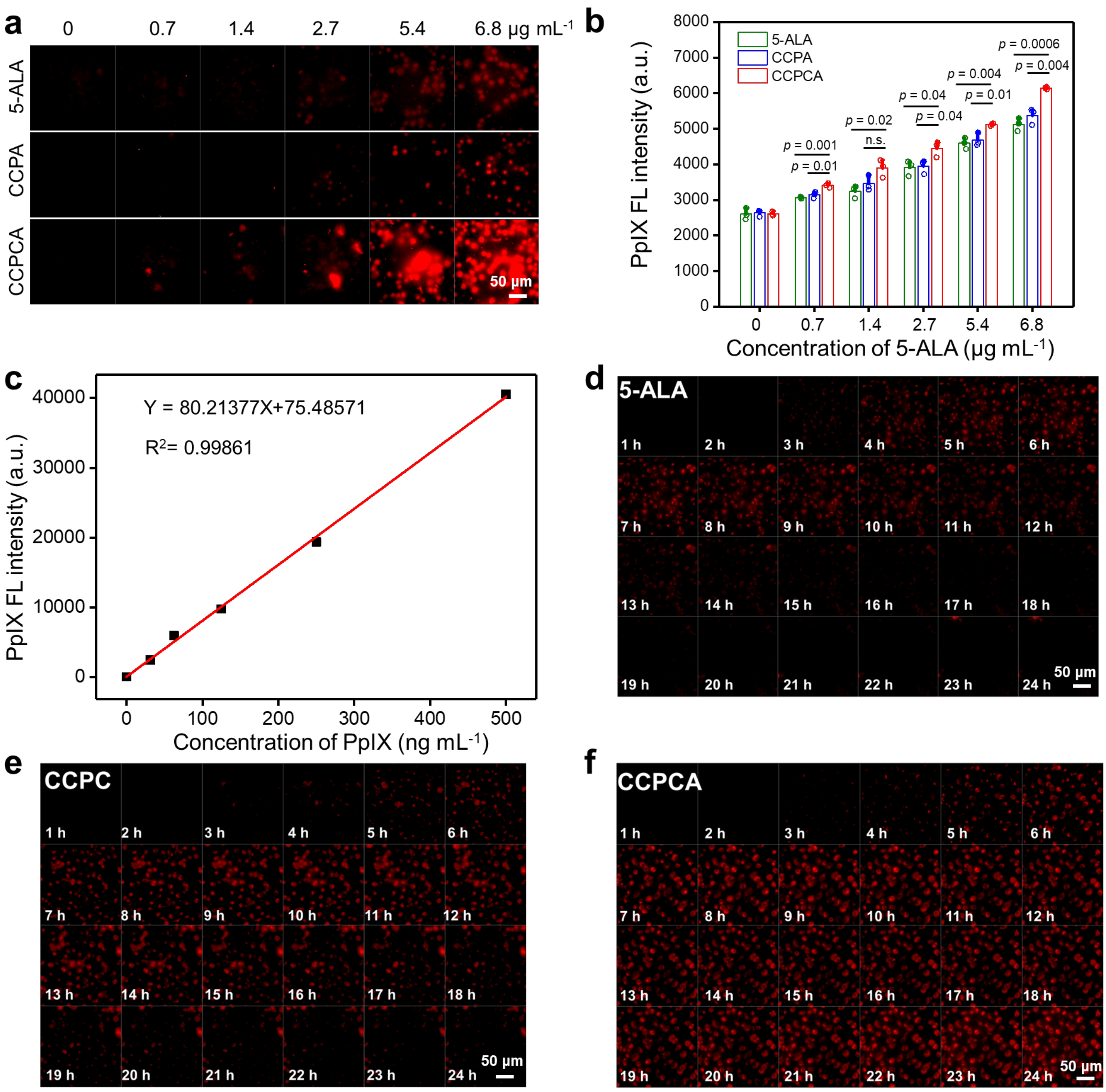

Supplementary Figure 6 CCPCA NPs maximize the accumulation of PpIX. (a) FL images of PpIX and **(b)** quantitative analysis of FL intensity of PpIX in A375 cells upon exposure to different concentrations of 5-ALA, CCPA and CCPCA NPs (equivalent 5-ALA) for 24 h, scale bar = 50 μm. Data represent the means ± SD (n = 3). Statistical significance was calculated via two-tailed Student's t test. **(c)** The standard curve of FL intensity of PpIX solutions (excitation wavelength = 400 nm, emission wavelength = 635 nm). n = 3 independent experiments. Real-time FL images of PpIX in A375 cells within 24 h after treatment with **(d)** 5-ALA (10.8 μg mL^{-1}), **(e)** CCPA and **(f)** CCPCA NPs (160 μg mL^{-1}), scale bar = 50 μm. Images are representative of seven biologically independent replicates (n = 7). Source data are provided as a Source Data file.

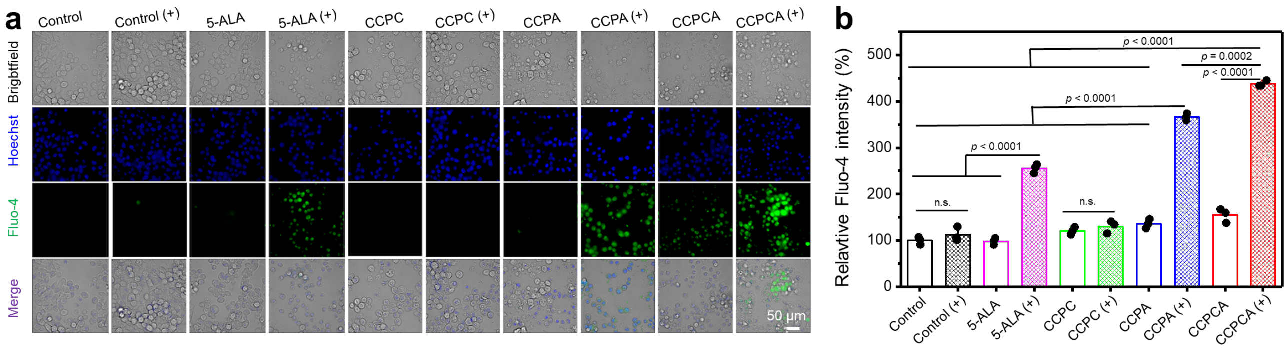

Supplementary Figure 7 Oxidative stress-mediated $[Ca]_{CYT}$ overload. (a) FL images of intracellular $[Ca]_{CYT}$ labeling by Fluo-4 AM and (**b**) corresponding quantitative analysis after incubation with blank DMEM, 5-ALA, CCPA and CCPCA NPs for 24 h and then irradiation with a 635 nm laser (200 mW cm^{-2}, 5 min) or not, scale bar = 50 μm. Data represent the means ± SD (n = 3). Statistical significance was calculated via two-tailed Student's t test. Source data are provided as a Source Data file.

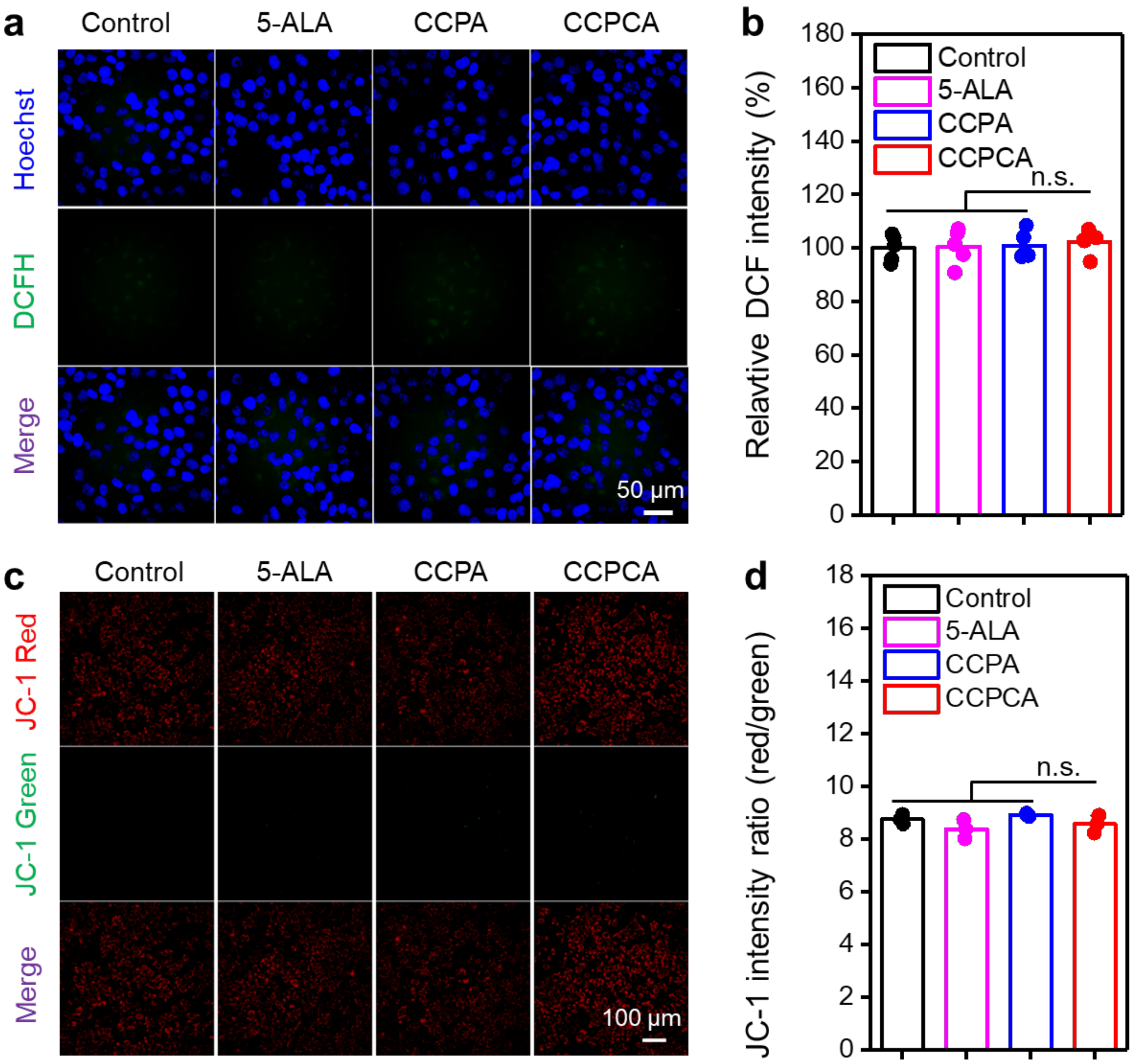

Supplementary Figure 8 Lack of laser irradiation cannot induce oxidative stress. (a) FL images and **(b)** quantitative analysis of DCF intensity after irradiation in different treatment groups of free-5-ALA, CCPA and CCPCA NPs (equivalent 5.4 μg mL^{-1} 5-ALA) for 24 h, scale bar = 50 μm. **(c)** FL images and **(d)** quantitative analysis of JC-1 aggregates (red) and monomer (green) intensity in the different treatment groups comprising control, 5-ALA, CCPA and CCPCA NPs (equivalent 5.4 μg mL^{-1} 5-ALA) after irradiation for 24 h, scale bar = 100 μm. All data represent the means ± SD (b, n = 5, d, n = 3). Statistical significance was calculated via two-tailed Student's t test. Source data are provided as a Source Data file.

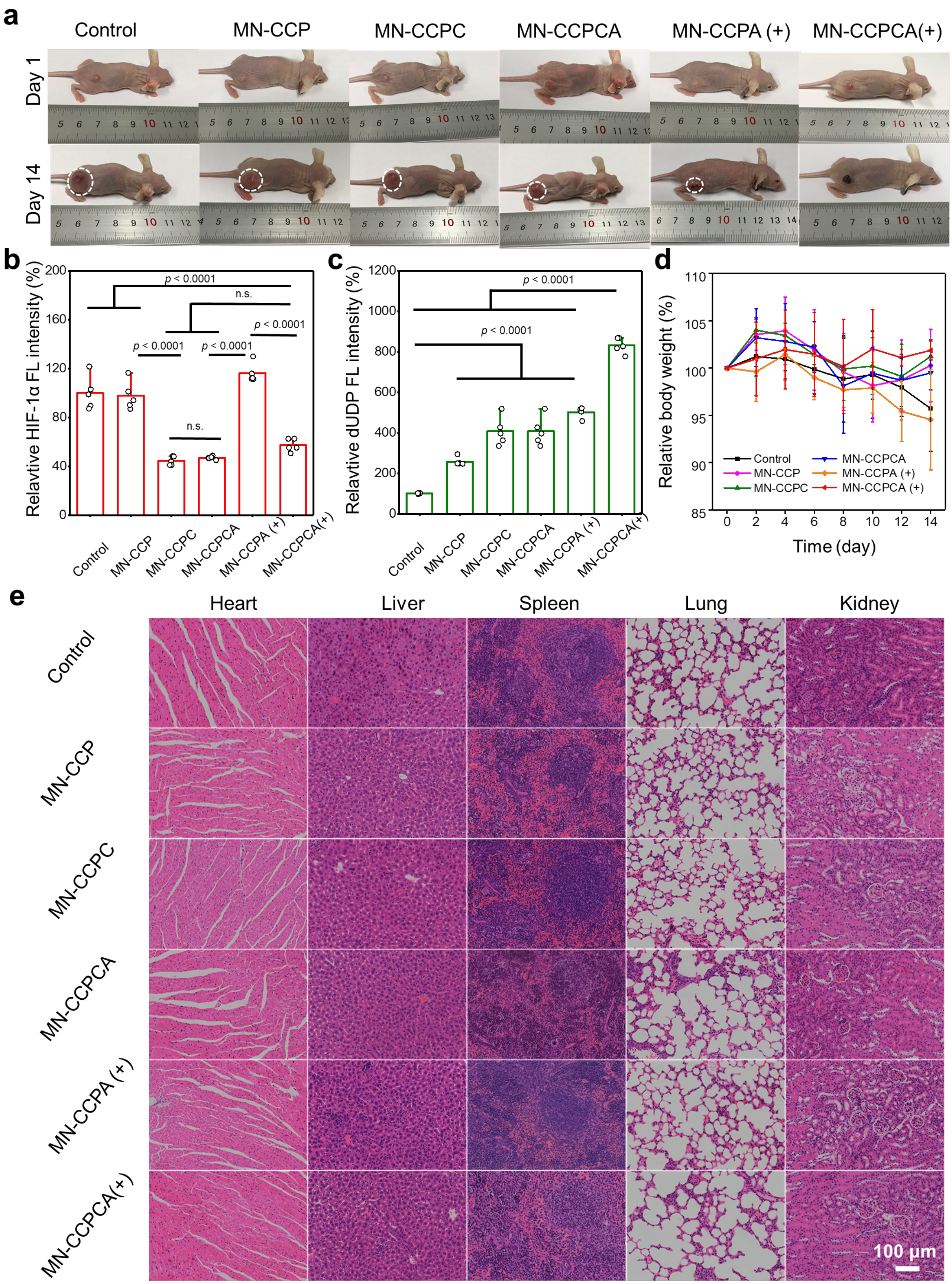

Supplementary Figure 9 Biosafety after the application of MN patches. (a) Photographs of A375 tumor-bearing mice before treatment (day 0) and at the end of treatment (day 14).

Images are from five biologically independent female A375 tumor-bearing mice, n = 5. Quantitative analysis of **(b)** HIF-1α **(c)** dUDP FL intensity in A375 slices after application of different MN patches. FL intensities were quantified using Image J. Data represent the means ± SD, (n = 5). Statistical significance was calculated with two-tailed Student's t test. **(d)** Relative body weight of A375 tumor-bearing mice during treatment. Data represent the means ± SD, (n = 5). **(e)** H&E staining images of main organs (heart, liver, spleen, lung and kidney) of A375 tumor-bearing mice after application of different MN patches for 14 days, scale bar = 100 μm. Images are from five biologically independent six-week-old female A375 tumor-bearing mice, n = 5. Source data are provided as a Source Data file.

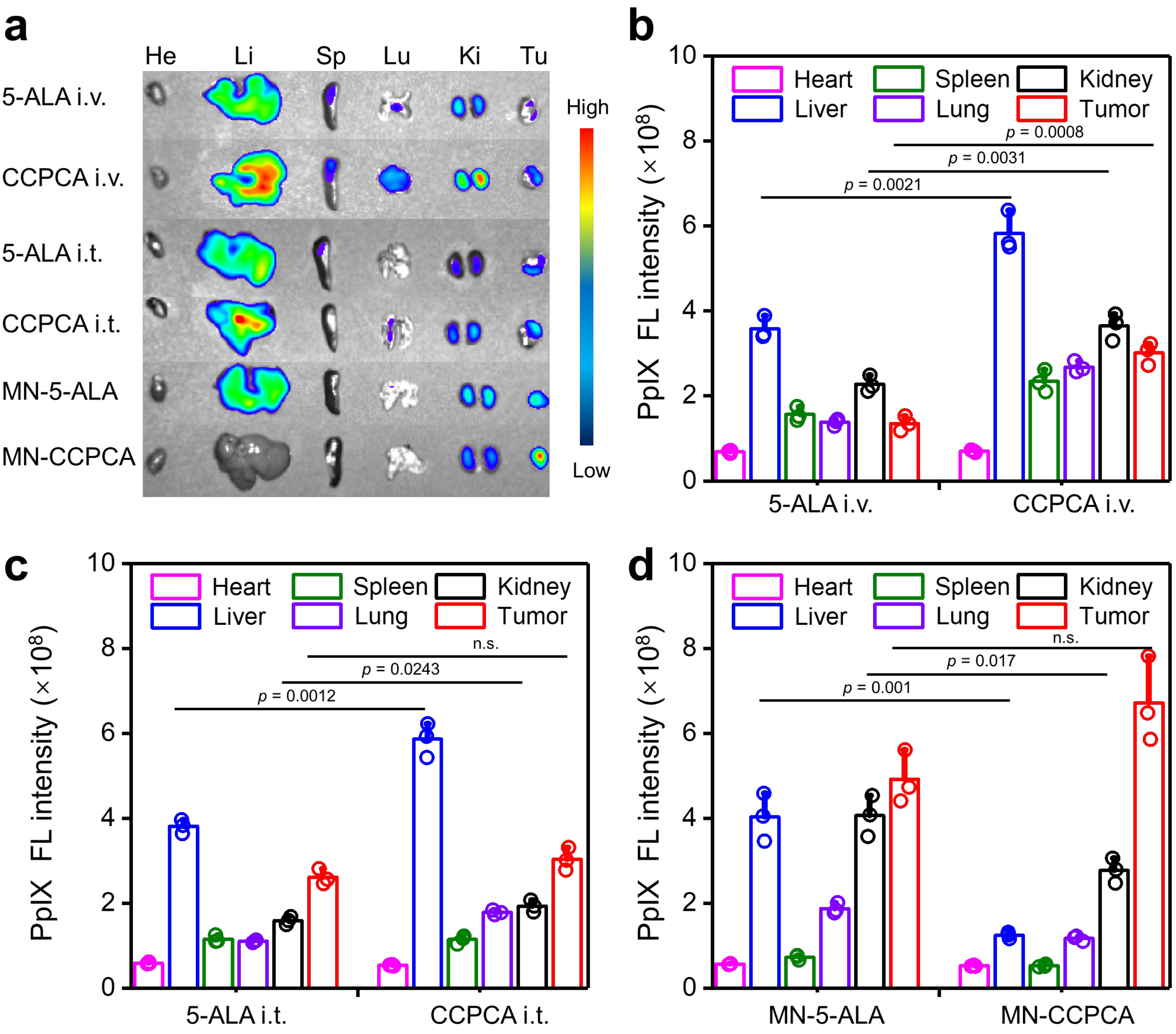

Supplementary Figure 10 Ex vivo FL images of major organs and tumors in 4T1 tumor model after different administrations of 5-ALA and CCPCA. (a) FL images of major organs and tumors of 4T1 tumor-bearing mice and quantification of FL intensity of PpIX after application of **(b)** 5-ALA i.v. and CCPCA i.v., **(c)** 5-ALA i.t. and CCPCA i.t., **(d)** MN-5-ALA and MN- CCPCA in major organs and tumors ex vivo. Images are from three biologically independent four-week-old female 4T1 tumor-bearing mice. Data are presented as mean ± SD, n = 3. Statistical differences were determined by Two-tailed Student's t test. Source data are provided as a Source Data file.

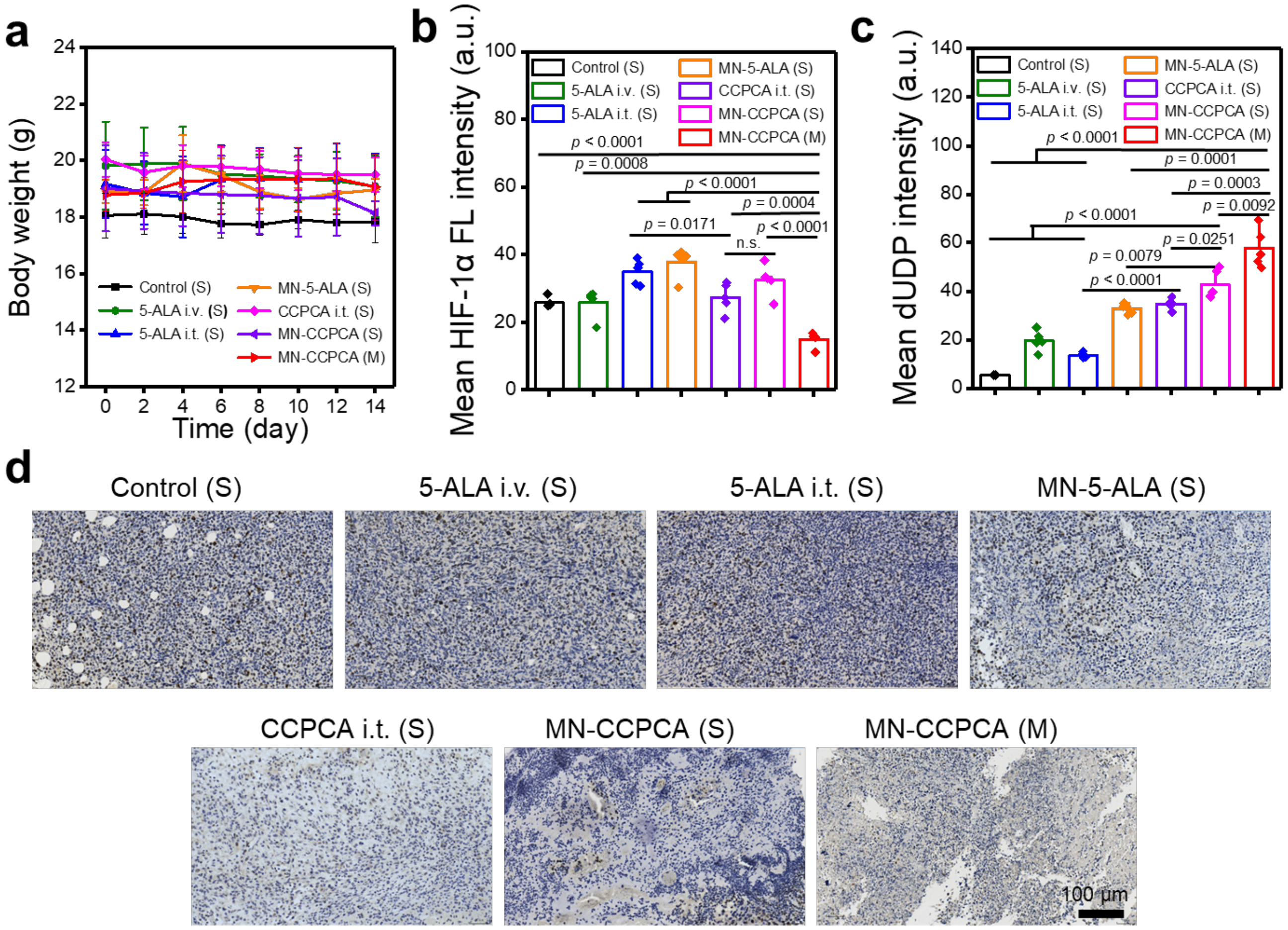

Supplementary Figure 11 Antitumor effects of MN-CCPCA patch in a 4T1 tumor model. (a) Body weight of 4T1 tumor-bearing mice in different treatment groups. Data represent the means ± SD, (n = 6). Quantitative analysis of **(b)** HIF-1α and **(c)** dUDP FL signals in 4T1 tumors. FL intensities were quantified using Image J. Data are representative of five biologically independent replicates, n = 5. **(d)** Ki67-stained sections of 4T1 tumors after various treatments. Images are from six biologically independent 4T1 tumors of six-week-old female BALB/c-nude mice, n = 6. Statistical significance was calculated with two-tailed Student's t test. Source data are provided as a Source Data file.

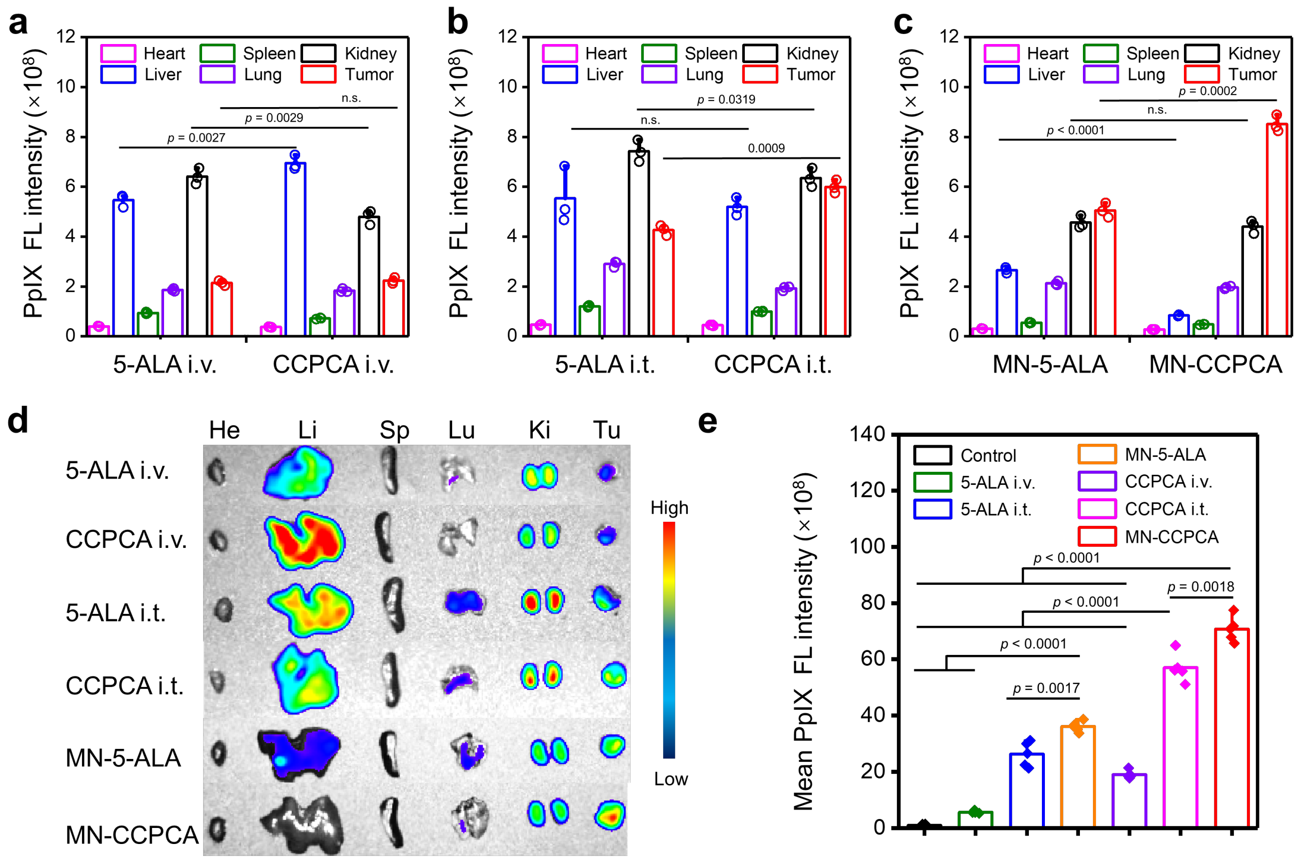

Supplementary Figure 12 Ex vivo FL images of major organs and tumors in U87MG tumor model after different administrations of 5-ALA and CCPCA. Quantification of FL intensities of PpIX after application of **(a)** 5-ALA i.v. and CCPCA i.v., **(b)** 5-ALA i.t. and CCPCA i.t., **(c)** MN-5-ALA and MN- CCPCA, and **(d)** corresponding FL image in major organs and tumors ex vivo. Images are from three biologically independent four-week-old female U87MG tumor-bearing mice. Data represent the means ± SD, (n = 3). **(e)** Quantitative analysis of PpIX FL signals in 4T1 tumors after various treatments. FL intensities were quantified using Image J. Data are presented as mean ± SD, (n = 5). Statistical differences were determined by Two-tailed Student's t test. Source data are provided as a Source Data file.

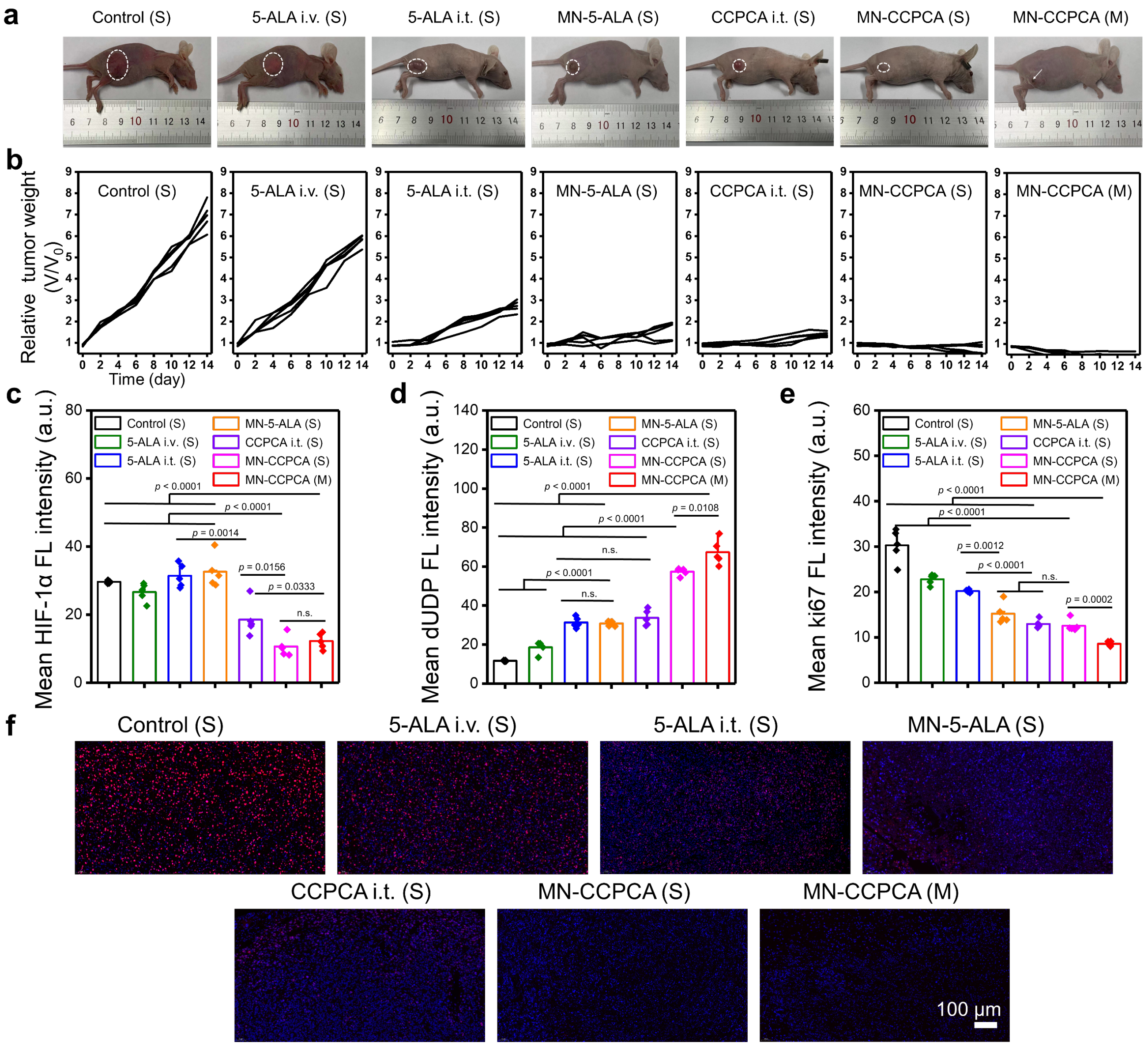

Supplementary Figure 13 Antitumor effects of MN-CCPCA patch in a U87MG tumor model. (a) Photographs of U87MG tumor-bearing mice at the end of treatment (day 14). Images are from five biologically independent U87MG tumors-bearing mice, n = 5. **(b)** Individual tumor growth curves of U87MG tumor weight in different treatment groups on day 14. Data are presented as mean ± SD, (n = 5). Quantitative analysis of **(c)** HIF-1α, **(d)** dUDP and **(e)** Ki67 FL intensity in U87MG tumor slices after different treatments. FL intensities were quantified using Image J. Data are representative of five biologically independent replicates, n = 5. **(i)** Ki67-stained sections of U87MG tumors after various treatments. Images are from five biologically independent U87MG tumors of six-week-old female BALB/c-nude mice, n = 5. Statistical significance was calculated with two-tailed Student's t test. Source data are provided as a Source Data file.

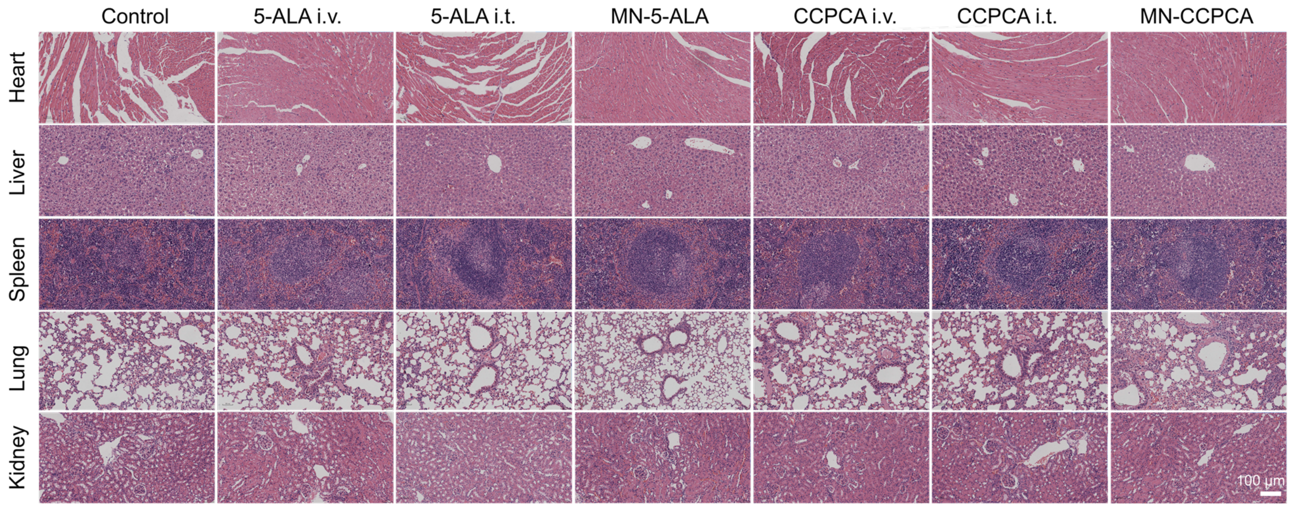

Supplementary Figure 14 H&E staining of tumor slices collected from different administration of 5-ALA and CCPCA groups. Main organs (heart, liver, spleen, lung and kidney) of U87MG tumor-bearing mice after various treatments was collected for H&E staining at 14th day, scale bar = 100 μm. Images are from three biologically independent six-week-old female U87MG tumor-bearing mice, n = 3. Source data are provided as a Source Data file.

Supplementary Table 1. Accurate calculated value of tumor area, positive area ratio and positive area density of anti-GSH FL signals in 4T1/U87MG tumors by Aipathwell immunofluorescence analysis.

	Tumor area (mm^2)		Positive area ratio (%)		Positive area density (a.u.)	
	4T1 tumor model	U87MG tumor model	4T1 tumor model	U87MG tumor model	4T1 tumor model	U87MG tumor model
Control (S)	69.7506	67.7756	18.53	17.93	0.0158	0.0188
ALA i.v. (S)	50.0448	39.9512	22.8	18.12	0.0148	0.0145
ALA i.t. (S)	31.3791	15.2056	18.73	15.5	0.0166	0.0114
MN-ALA(S)	25.9586	12.9857	15.75	12.5	0.0133	0.012
CCPCA i.t. (S)	20.987	12.2066	7.5	1.81	0.0073	0.0068
MN-CCPCA (S)	8.5373	7.9057	2.18	1.38	0.0011	0.0049
MN-CCPCA (M)	7.3306	5.2153	0.31	1.05	0.0003	0.0011
